# Leveraging H1N1 infection transmission modeling with proximity sensor microdata

**DOI:** 10.1186/1472-6947-12-35

**Published:** 2012-05-02

**Authors:** Mohammad Hashemian, Kevin Stanley, Nathaniel Osgood

**Affiliations:** 1 Department of Computer Science, University of Saskatchewan, Saskatoon, Canada; 2 Department of Community Health and Epidemiology, University of Saskatchewan, Saskatoon, Canada

## Abstract

**Background:**

The contact networks between individuals can have a profound impact on the evolution of an infectious outbreak within a network. The impact of the interaction between contact network and disease dynamics on infection spread has been investigated using both synthetic and empirically gathered micro-contact data, establishing the utility of micro-contact data for epidemiological insight. However, the infection models tied to empirical contact data were highly stylized and were not calibrated or compared against temporally coincident infection rates, or omitted critical non-network based risk factors such as age or vaccination status.

**Methods:**

In this paper we present an agent-based simulation model firmly grounded in disease dynamics, incorporating a detailed characterization of the natural history of infection, and 13 weeks worth of micro-contact and participant health and risk factor information gathered during the 2009 H1N1 flu pandemic.

**Results:**

We demonstrate that the micro-contact data-based model yields results consistent with the case counts observed in the study population, derive novel metrics based on the logarithm of the time degree for evaluating individual risk based on contact dynamic properties, and present preliminary findings pertaining to the impact of internal network structures on the spread of disease at an individual level.

**Conclusions:**

Through the analysis of detailed output of Monte Carlo ensembles of agent based simulations we were able to recreate many possible scenarios of infection transmission using an empirically grounded dynamic contact network, providing a validated and grounded simulation framework and methodology. We confirmed recent findings on the importance of contact dynamics, and extended the analysis to new measures of the relative risk of different contact dynamics. Because exponentially more time spent with others correlates to a linear increase in infection probability, we conclude that network dynamics have an important, but not dominant impact on infection transmission for H1N1 transmission in our study population.

## Background

The threat of emerging infectious diseases has stimulated the search for techniques to prevent and control communicable disease spread [1]. Simulation models have emerged as key tools in examining trade-offs between multiple health interventions, and in aiding the control of communicable diseases [2]. While properly parameterized and calibrated models can inform decision making, building such models is challenging because critical parameters are difficult to measure precisely, including the structure and dynamics of contact networks among population members, which shape the spread of both pathogens and risk behaviors.

Data collected by contact tracing [3] and self-reporting [4] has provided some important insights into network structure for many notifiable illnesses. Unfortunately, even for the best models, contact data depends heavily on unreliable self-reporting data collection methodologies [5] which omit much detail and place a substantial recording burden on participants [4]. Because of the less tangible character of contacts involved, determining contact network structure for air-borne pathogen spread requires collection of additional information on casual contacts [4]. While self-reported measures can provide insight, some leading studies have noted the desirability of employing automated data-collection approaches to capture higher fidelity contact frequency and duration information [4].

Some early work in the linking of health and micro-contact data has been reported [6,7]. The work of [6], with their single-day tracking of several hundred high-school students, forms a particularly important basis and methodological framework for the integration of micro-contact data and disease simulation models. However, in both these cases, limited health information was collected and a stylized infection model was utilized to extract the impact of contact network dynamics on the spread of infection.

As the first influenza pandemic in decades, the H1N1 pandemic – whose initial outbreak was described in April 2009 – served as a catalyst for research into control of emerging infectious diseases. Within the study site of Saskatoon (a Midwestern Canadian city of approximately 250,000 people) H1N1 first emerged in Spring 2009, and followed the typical summer quiescence, and Autumnal re-emergence. By mid-October, cases of H1N1 began a notable rise [8]. At the same time, vaccination initiated in a staged fashion. Mass vaccination proceeded aggressively from early November through December 18. Vaccination data suggest that approximately 50% of the city population was vaccinated [9]. Aided by the staged vaccination process, reported cases of influenza in 2009–2010 peaked unusually early (mid-November). Low numbers of influenza cases were reported in December 2009 and thereafter. Most circulating influenza transmission in Saskatchewan over this period was drawn from the H1N1 strain [8].

In anticipation of the significance of the 2009–2010 influenza season, the co-authors had launched a previously-described [10] pilot study in the City of Saskatoon to electronically collect contact patterns between 36 participants in addition to their influenza-related health status information. Each participant was requested to carry a proximity sensor at all times during the study period, as well as to fill out a sequence of weekly health surveys via a web browser. The study started on November 9th 2009 and finished on February 9th 2010, collecting all contacts between 36 individuals for 92 days. It recorded a total of approximately 265,000 thirty-second proximity time slots between individuals cross-linked to weekly self-reported health status and contact history.

In this work, we sought to integrate rich contact micro-data collected in [10] with an adaptation of a well-grounded individual-level Canadian transmission model [11], and population-level statistics on the infection rates for the province where the outbreak occurred in an agent-based model. Our study objectives were threefold: to assess the effectiveness of incorporating contact micro-data with models of infectious disease, to identify features within empirical contact patterns that exerted disproportionate impacted on infection spread, and to validate these findings against self-reported health status information.

Unlike [6] we opt for a smaller study population (36 as opposed to 788) but much longer duration (92 days as opposed to a single day) allowing us to evaluate the evolution of contact patterns and disease with the health state of the individuals throughout the flu season. Using collected health data, we can compare the results of our simulation to the infection rate in the province and amongst the participants. In this paper we make the following contributions:

1. A novel methodology for integrating disease, population level, and micro-contact data into a coherent agent-based simulation framework, validated by comparison with the actual health status of the study population;

2. A comparison of metrics for measuring the risk associated with contact and contact-duration, culminating in a novel measure: log time-degree (LTD);

3. A demonstration of the utility of micro-contact data during an epidemic outbreak based on both empirical and simulation results;

4. A preliminary investigation into the role of dynamic network structure on the spread of disease, and the impact of vaccination on that structure.

## Methods

**Figure 1:**
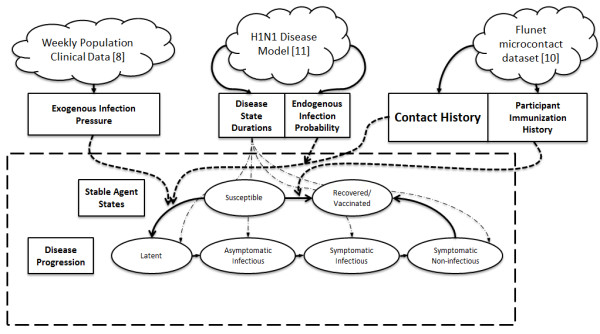
Simulation structure and flow.

Our primary data source was Flunet [10]. While other contact datasets are available [6,12], and others with health information have been described [13], Flunet is unique in providing longitudinal information on contact patterns, occurrence of influenza-like illness (ILI) symptomology, and vaccination status obtained during an epidemic outbreak in the region under observation. Our micro-contact data contained detailed inter-participant contact patterns but lacked data about the threat of infection from non-participants, which we modeled using reported H1N1 incidence data for the same time and province as the contact data [8]. A system diagram outlining the flow of data and state changes is shown in Figure 1.

The simulation model is encapsulated in the dashed box. Agents remained in a susceptible state unless acted on by an infection event. Such stochastic events were triggered externally using the exogenous pressure data derived from case reports [8], or internally through contact with an infected agent. The likelihood of endogenous infection was governed by contact and vaccination data from [10] and infection risk information drawn from [11]. Because we assumed H1N1 re-infection risk to be negligible, agents in the recovered or vaccinated state remained there until the simulation ended. If an agent became infected, the infection ran its course deterministically through the stages of infection. The duration of each infection stage was drawn from the distributions in [11] and derived quantities.

We performed Monte Carlo ensembles of stochastic dynamic simulations operating on the contact data, where the primary variables drawn from distributions were disease stage durations, exogenous infection event rates and the probability of transmission from infected endogenous contacts. Ensembles were selected in a memoryless fashion based only on the disease parameters. In every realization (simulation run), the contact record was stepped through like an animation, creating exactly the same sequence of contacts in the course of every Monte Carlo realization, which we have termed a Groundhog Day technique, after the 1993 movie of the same name. This is similar to the technique we employed in [7], but different from the technique employed by [6] where a single daily aggregate contact network was employed. While our technique is computationally more expensive, it captures inter- and intra-day heterogeneity in the contact network. While agents repeatedly relived the same sequence of contacts in different realizations, the stochastics associated with infection progression and transmission gave rise to differences in disease spread.

### Contact data

The Flunet study population consisted of 36 participants, each carrying a small wireless sensor (or “mote”) capable of short-range wireless communication [10]. Participants were asked to carry the sensors with them at all times during the experiment period. In addition, 11 stationary motes were deployed at fixed high-traffic locations, picked by experimenters, in order to study the contact patterns between people and places [14]. Three of these stationary motes were also connected to a networked PC and acted as data sinks, opportunistically receiving accumulated data from nearby mobile motes.

When two motes were in close proximity, they would each record a contact with a minimum resolution of 30 seconds. Each contact record represented a contact session between two motes, which included the start and end time of a contact, and the distance between the adjacent motes. A contact’s distance was estimated by binning the received signal strength indicator (RSSI, a measure of the wireless signal strength) into close (< 5 m), medium (5–15 m) and far (>15 m) bins [10]. Although contacts between participants and stationary nodes at various ranges were recorded, only close contact data from person-person interactions are considered here. Participants were asked to fill out a sequence of weekly health surveys which included symptoms and diagnoses of ILI, reported date of H1N1 vaccination, and self-reported contact patterns. Demographic data was collected in a single survey at the conclusion of the study.

**Figure 2:**
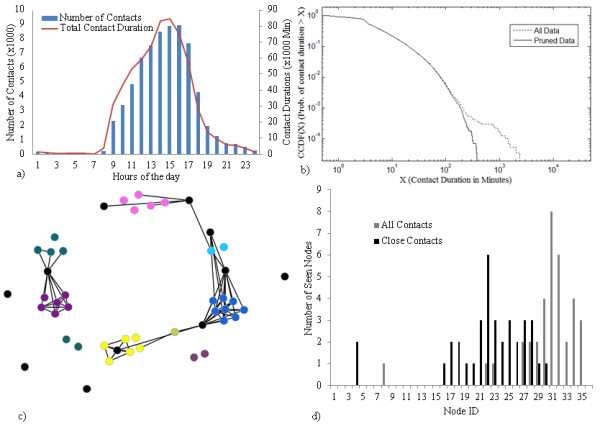
**Flunet Findings.a)** Contact histogram by hour of day, **b)** CCDF of contact duration, **c)** Connectivity graph with threshold of 18 minutes per day average contact. Black nodes represent stationary nodes associated with a location, and are included in this graph for illustrative purposes only. **d)** Network span for close and all contacts.

A preliminary analysis of the dataset is provided in [10]. Analysis in Figure 2a and 2b has been reproduced here for the readers’ convenience while additional analysis germane to the application of the dataset to agent-based modeling of infectious disease (Figure 2c and 2d) has been added.

Figure 2a shows that contacts are tightly clustered throughout the workday, with staff arriving in the morning and graduate students trickling in throughout the day. Sporadic contacts are recorded throughout the evening and night. This figure also illustrates that the contact data contains primarily workplace relationships. Figure 2b shows the complementary cumulative distribution function (CCDF) of contact duration. In addition to the CCDF for all the collected data (solid line), we removed contacts with durations exceeding 7 hours (0.03% of total reported contacts) from the raw dataset (dashed line) because we assumed contact of this duration was due to sensors abandoned near each other. Removing this section of data yields considerable differences in the distribution’s tail. The CCDF is broadly consistent with other long-term datasets of this nature [15]. The heterogeneity of the contact distribution is important for our hypotheses and assumption - that contact dynamics have significant impacts on infection rate as initially noted by [6] – which in turn drove the simulation design. In Figure 2b, contact duration spans more than two orders of magnitude. If we posit that an infectious individual gives rise to contagious events (e.g., sneeze or cough) with some stochastic arrival probability independent of the contact duration, a susceptible is likely to experience more contagious events in a prolonged contact than in a shorter one, a property assumed in some other modeling studies [16].

To visually highlight the impact of cliques and place on the dataset, Figure 2c plots the relationship between contacts which existed for an average of 18 min/day. This threshold was to represent a plausible amount of time per day that a regular contact might have occurred over the course of the study, bearing in mind that weekends and holidays are included in the denominator. Black nodes represent stationary nodes associated with a location, and are included in this graph for illustrative purposes only. As is apparent in the graph, nodes that are generally collocated have a high degree of contact with each other. Nodes that are not collocated have much lower connectivity, with the exception of a few bridging individuals.

Given the importance of network structure, we consider the span of the network in Figure 2d, which is closely related to degree centrality (see Network Metrics Section). This graph is shown for two scenarios: a scenario where only close proximity constitutes a contact, and a scenario where any detectable presence qualifies. When limiting the analysis to close contacts, the histogram is both more peaked and has a lower mean than when considering all the possible contacts. The modes are 22 and 31, respectively, implying that many participants saw most of the other participants at least once. However, because it is not saturated at the maximum (as would be the case if all participants saw all other participants) it is logical to hypothesize that some partially isolated cliques exist, and that the close contact network is more strongly cliqued.

### Transmission model

#### Model design

The simulation model classified each individual in the sample population into one of seven states: *Susceptible*, *Latent*, *Asymptomatically Infectious*, *Symptomatic Infectious*, *Symptomatic Non-Infectious*, *Recovered*, and *Vaccinated*. All the agents in the model started in the *Susceptible* state, consistent with limited pre-existing population immunity to H1N1. A susceptible individual could contract the infection either from exogenous or endogenous sources. Exogenous sources are defined as the population outside the study who were in contact with Flunet participants and could transmit the infection to the monitored individuals, while endogenous sources are other Flunet participants in an infectious state.

Dynamic transmission models differ in their treatment of contacts. For some epidemiological contexts, the contacts underlying transmission are of defined or bounded duration – for example, needle sharing, sexual encounters, and blood transfusions. For this class of contacts, the frequency rather than the duration of contacts is the primary source of variability in transmission risk. For air-borne infections, however, the likelihood of transmission rises not only with contact frequency, but also with contact duration [6,16].

For the case of H1N1 influenza transmission, our model assumes that on-going contact between two discordant individuals provides a conduit for transmission, where the likelihood density of transmission is a constant independent of contact duration. More specifically, we posit that an infectious individual gives rise to potentially contagious events (e.g., sneeze or cough) at a fixed rate *v*. We further treat any susceptible individual in contact with that person as having a likelihood *β* of contracting the infection for each such infectious event. Similar to the analysis of [6,16], infections are more likely to occur in a longer contact than in a shorter one.

Given this model, the basic reproductive number *R*_*0*_ (the average number of secondary infections caused by an infective individual, in an otherwise susceptible population) is as follows:

(1) $$R_{0}=\bar{T_{i}}\bar{F_{C}}v\beta$$

where $\bar{T_{i}}$ is the mean duration of infectiousness in days, $\bar{F_{c}}$ represents the average daily cumulative contact duration of an individual in time slots (summed regardless of concurrency), *v* is the number of potentially infecting events per time slot of contact between two individuals, and *β* is the mean likelihood of a susceptible will be infected by a given infecting event.

For endogenous infections, we assumed that the mean of the basic reproductive number R_0_ for our study population was equal to 1.31, identical to that reported in a prominent Canadian H1N1 study [11]. For an infective person, the infection hazard of infecting an adjacent susceptible individual per time step (*βv*) was thus determined as follows:

(2) $$\beta v=\frac{R_{0}}{\bar{T_{i}}\bar{F_{c}}}$$

Where *T*_*i*_ is set to 3.38 [11], and $\bar{F_{c}}$ is computed using recorded data in Flunet dataset according to the following formula:

(3) $$\bar{F_{c}}=\frac{\sum_{i=1}^{N_{p}}\sum_{j=1}^{N_{p}j\neq i}T_{c}\left(i,j\right)c}{N_{p}N_{d}}$$

where *N*_*p*_ represents the number of study participants, *N*_*d*_ gives the study duration, and *T*_*c*_(*i*, *j*) indicates the total duration of contacts (in days) between nodes *i* and *j* during *N*_*d*_ days.

**Figure 3:**
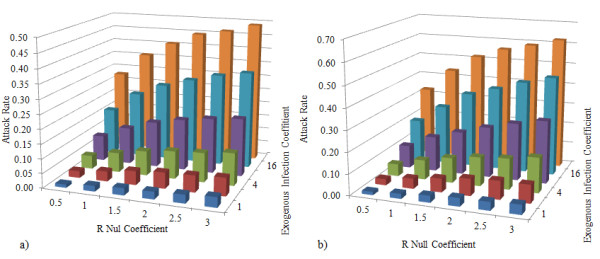
Weekly laboratory-confirmed H1N1 cases reported in Saskatchewan.

The per-week infection hazard of acquiring the infection from exogenous sources was determined according to the following formula:

(4) $$P_{x}(k)=\frac{N_{k}}{N_{U}-\sum_{i=1}^{k-1}N_{i}}$$

where *k* refers to the week number since the start of the simulation, *N*_*i*_ gives the number of laboratory confirmed H1N1 cases in the province of Saskatchewan during the *i*^*th*^ week of the 2009–2010 influenza season, and *N*_*U*_ refers to the total population of the province. The denominator represents an estimate of the number of susceptible individuals in the province. The entire denominator thus estimates the number of susceptible individuals who remain at risk in week *k*. We note here the computation of susceptible individuals in the denominator consider neither the vaccination status of the population (for which reliable data was not available at the time of simulation), nor the infections that took place during the previous influenza season. This formulation therefore systematically underestimates the actual infection pressure. We compensate for this systematic error by providing a thorough exploration of the space of possible transmission probabilities (Figure 3).

A susceptible agent receiving the infection from either exogenous or endogenous sources transitions to the latent state. Before starting the Latent period, the model computed the duration for each of the subsequent four stages of illness (Figure 1). In determining these durations, we sought to reproduce the observed variability in H1N1 progression by drawing the duration of incubation (*T*_*Inc*_) and duration of symptoms (*T*_*S*_) from two log-normal distributions with parameters from [11]. The illness duration *T*_*Ill*_ was calculated by adding *T*_*Inc*_ and *T*_*S*_. Using these three values, the total duration of infectiousness *T*_*Inf*_ was calculated as:

(5) $$T_{\mathrm{Inf}}=\frac{T_{\mathrm{Ill}}\bar{T_{\mathrm{Inf}}}}{\bar{T_{\mathrm{Inc}}}+\bar{T_{S}}}$$

where *T*_*Ill*_ gives the computed duration of illness, $\bar{T_{\mathrm{Inf}}}$ is the average duration of infectiousness, $\bar{T_{\mathrm{Inc}}}$ shows the average incubation period, and $\bar{T_{S}}$ is average duration of symptoms. Following the computation of the duration of infectiousness, the duration of the *Symptomatic Infectious* state *T*_*sInf*_was estimated using:

(6) $$\frac{T_{\mathrm{sInf}}}{T_{\mathrm{Inf}}}\approx \frac{\bar{T_{\mathrm{sInf}}}}{\bar{T_{\mathrm{Inf}}}}$$

The remainder of the durations were computed using following subtractions:

(7) $$T_{\mathrm{aInf}}=T_{\mathrm{Inf}}-T_{\mathrm{sInf}}$$

(8) $$T_{\mathrm{lat}}=T_{\mathrm{Inc}}-T_{\mathrm{aInf}}$$

(9) $$T_{\mathrm{nInf}}=T_{S}-T_{\mathrm{sInf}}$$

where *T*_*aInf*_ represents the asymptomatically infectious duration, *T*_*lat*_ shows the latent period duration, and *T*_*nInf*_ shows the symptomatic non-infectious duration.

Each infected agent experienced the four illness states sequentially with the passage of time. A person in the *Asymptomatically Infectious* or *Symptomatic Infectious* state was considered infective, and could infect other susceptible adjacent individuals with infection hazard *βv*. At each time step and for each adjacent susceptible, the infective person transmitted the infection with likelihood density *βv*. A susceptible receiving H1N1 vaccination transitioned to the *Vaccinated* state, and was thereafter considered immune.

For the sake of the simulation, we assumed no H1N1 mortality. Our study lacked sufficient data to predict whether a specific individual would elect to self-quarantine given a symptomatic infection, and did not consider hospitalization outcomes. Given these assumptions, we chose to regard an individual’s contact patterns as unaffected by the health status of that individual and those around them. To examine the degree to which these assumptions might shape simulation results, we simulated an additional Monte Carlo ensemble examining the extreme situation in which individuals removed themselves from circulation for the duration of their symptomatic period. Finally, in light of the dominance of the H1N1 strain during the Saskatchewan 2009–2010 influenza season, only one strain of influenza was considered.

#### Simulation setup

The model described in the previous sections was implemented in Network Simulator 3 (ns-3), a discrete-event simulator. A network of 36 agents was created, where each agent represented one individual. The Flunet study data was discretized into 30-second time slots, and at each time slot the connectivity between each pair of individuals was updated based on the contacts recorded in the Flunet dataset. This dynamic contact network can be visualized as a time-varying graph where edges appear or disappear every time step depending on whether the two participants were in contact. The network could also be effectively encoded as a fully connected graph where edge weights at every time step have a value of 0 (unconnected) or 1 (connected). The fully connected graph representation can easily be implemented as a time series of sparse symmetric matrices (one for every time step) where 0 represents no connection between the node (*i,j*) and 1 represents a connection. A variant of this representation of the connectivity pattern was employed. A flat text file for each agent was created containing a vector representing the contact between an agent and all other agents for every time step in the experiment. Before starting a realization, each agent loaded the connectivity file, and at each time step referenced the appropriate vector (line in the file) to infer its connectivity with other agents at a given time.

To estimate *P*_*x*_, the model required a time series of the laboratory-confirmed H1N1 cases in Saskatchewan. This data was extracted from the Public Health Agency of Canada FluWatch [8] on a weekly basis. As it is shown in Figure 3, the number of reported cases for the 13 weeks of the simulation declines monotonically to zero after the 9^th^ week (January 4^th^ 2010), and therefore *P*_*x*_ in the model for 10^th^ week to the end of the simulation was zero.

**Figure 4:**
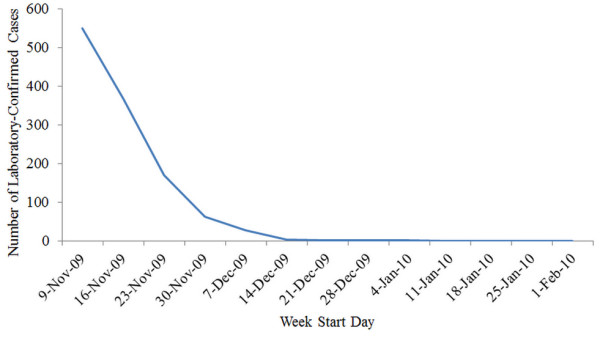
**Observed attack rate based on endogenous and exogenous infection pressure.** Attack rate (fraction of endogenous population infected) according to different assumptions about endogenous and exogenous infection pressure. In the left-hand panel, vaccination effect is incorporated, while in the right hand panel no vaccination is considered.

Each susceptible agent drew from a distribution at each time-step to determine whether it was infected by exogenous sources. If it contracted the infection – whether from exogenous or endogenous sources – the agent determined the integral duration (in units of time-steps) for each state of the infection based on the equations explained in Model Design section, and proceeded to remain each state for the determined number of time-steps. During the infectious period, the agent drew from a distribution to to determine whether it infected other nearby susceptible agents.

#### Scenarios

The simulation explored a three-dimensional scenario space that examined the impact on model outputs of four distinct assumptions. The first two assumptions related to the exogenous and endogenous forces of infection (FOI). An exogenous FOI coefficient linearly scaled *P*_*x*_ to values that were 1, 2, 4, 8, 16, and 32 times the baseline. Similarly, the endogenous FOI coefficient scaled *βv* by 0.5, 1, 1.5, 2, 2.5, and 3 times the baseline value.

The third assumption varied was whether the H1N1 vaccination status from [10] was considered during simulation. For the case without H1N1 vaccination, none of the self-reported H1N1 vaccination data was considered. For the scenarios that account for H1N1 vaccination, participants started susceptible but transitioned to the *Vaccinated* state according the time they reported an H1N1 vaccination in Flunet health surveys. Individuals who did not report vaccination in the surveys never entered the *Vaccinated* state. We assumed a negligible benefit of vaccination if the agent was infected at the time of vaccination, and allowed the infection to run its course.

One supplementary baseline scenario explored the impact of participants removing themselves from circulation during their symptomatic period. Note that to compute *βv* for this scenario, $\bar{T_{i}}$was replaced with $\bar{T_{\mathrm{aInf}}}$, where $\bar{T_{\mathrm{aInf}}}$ was calculated as the average duration of asymptomatically infectiousness of all the previous baseline and alternative scenarios.

In total, the scenario space consisted of three baseline scenarios and 72 additional scenarios. Each baseline scenario was simulated using 100,000 Monte Carlo realizations; the other 72 alternative scenarios were each simulated using 2,500 Monte Carlo realizations. Exploration of the scenario space (including the baselines and alternative scenarios) required running 480,000 different realizations.

#### Metrics for contact networks structure

While static representations of social networks are convenient, popular, and can yield powerful insights [17-20], the temporal aggregation involved may obscure features of real contact networks that serve important roles in the transmission of infectious disease. Our experimental and simulation design provided us with rich information to study network dynamics. However, because network structure – particularly evolving network structure – is difficult to represent concisely, derivative measures are often employed [19]. To quantify the structure of our network, we employed four centrality measures: betweenness, degree, time degree, and log time degree.

Betweenness centrality is a classic measure of network structure that attempts to capture the importance of the node to the graph’s connectivity, by summing the number of times a node lies on the shortest path between two other nodes, calculated using:

(10) $$C_{B}(v)=\sum_{a\in Nodes}\sum_{b\in Nodes-\{a\}}\frac{\Sigma_{\mathrm{ab}}(v)}{\Sigma_{\mathrm{ab}}}$$

where *v* is the vertex in question, *Σ*_*ab*_ is the number of shortest paths between *a* and *b*, and *Σ*_*ab*_(*v*) is the number of shortest paths between vertices *a* and *b* that pass through *v,* summed over all pairs of vertices in the graph.

While betweenness captures a global picture of the network by examining shortest paths, degree centrality only considers a node’s number of one-hop neighbors. For a static graph, degree centrality is calculated according to:

(11) $$C_{D}(v)=\frac{\deg(v)}{n-1}$$

where deg (*v*) is the number of edges incident on *v*, and *n* is the number of nodes in the graph. Degree centrality can capture the local conditions of a node more accurately, but does not take into account the heterogeneous nature of the contact patterns and durations evident in our dataset. As a result, we defined two additional centrality measures to capture ∈s of network dynamics.

Time degree centrality (TD) for a node can be defined as the average over all time slots of the fraction of all other agents with whom that node is in contact in a given time slot (analogous to the “strength” metric proposed in [6]). This is computed in our case by summing up the count of that node’s contacts over all 30-second time slots in the entire study, and then dividing by both the number of time slots in the whole study and by the number of participants − one. This measure captures the duration of (potentially concurrent) interpersonal contact patterns. People who encounter many others for short periods would have a large degree centrality, and a similar TD centrality to individuals who spend a great deal of time in a smaller group and have a smaller degree centrality. Because time degree aggregates over contact times, and because contact times are often characterized by exponential or power law distributions Contact Data Section [6,10,15], we also introduce log time degree (LTD) centrality as a measure of contact density. TD and LTD centralities are calculated discretely as:

(12) $$C_{\mathrm{TD}}(v)=\frac{1}{N_{k}}\sum_{k}C_{D}(v,k)$$

(13) $$C_{\mathrm{LDT}}(v)=\ln(C_{\mathrm{TD}}(v))$$

where *N*_*k*_ is the total number of time slots in the period and *C*_*D*_*(v,k)* is the degree centrality of the *v*^*th*^ vertex at time *k*. The LTD is simply the natural logarithm of the time degree. Time degree is normalized and therefore always less than or equal to one, causing log time degree to be always negative, with more negative numbers indicating a lower centrality.

If the heterogeneity of the system is dependent on the network structure, then the likelihood of a participant’s infection at some point during the study should be correlated with appropriate network structure metrics. We ran Pearson and Spearman correlations using the MATLAB statistical toolbox against the probability of infection in two baseline simulations (with and without vaccination) against the four measures of centrality described above. Given that an individual’s network location may also shape their likelihood of transmitting a pathogen when infected, for the same scenarios as above we ran correlations of the four centrality measures against the average number of secondary endogenous infections directly caused by a node once it was infected. Finally, to better understand the effect of vaccination status on the correlations derived above, we also used Student’s *t*-test to examine the difference in the four measures of centrality between participants who did and who did not report vaccination.

## Results

We analysed the response of our simulated infections to changing endogenous and exogenous infection pressure and the proximity threshold required for transmission to confirm that the simulation did not produce any significant artefacts. This served both as a cross-check on the H1N1 influenza model proposed in [11] using highly detailed contact data, and as a confirmation of the plausibility of our model and approach. With plausibility of approach established, we then used the baseline scenarios to examine the impact of network dynamics on the spread of infectious disease.

### Transmission model

Figure 4 shows the attack rate (fraction of the simulated study population infected) for 72 different scenarios, where vaccination effects were and were not considered. Figure 4a shows the attack rate for simulations that considered the effect of vaccination, and resulted in attack rates from 0.01 to 0.49. Figure 4b shows the graph of scenarios where no immunization through vaccination allowed, yielding an attack rate ranging from 0.02 to 0.62.

Self-reporting of participants’ health conditions in the Flunet dataset [10] indicated that one individual was diagnosed with influenza by a physician and two others reported symptoms characteristic of ILI (2.7% and 8.3% of the study population, respectively). Given that the parameters related to exogenous and endogenous pressures in this model are derived based on laboratory-confirmed cases, we compared model results for H1N1 infections to the single physician-diagnosed case. Because of the stochastic ∈s in the model, the number of H1N1 cases occurring in a model scenario varies from realization to realization. The statistics from baseline simulations incorporating vaccination effects give a simulation mean of 0.39 for H1N1 case counts. 82.14% of realizations yielded no infections within the study population; 10.26% of realizations contained exactly one infection; 7.6% of realizations yielded 2 or more infections. As the observed count of 1 person infected falls readily within the 95% empirical fractile around the mean, the null hypothesis that our model is consistent with the underlying epidemiological process cannot be rejected. However, we are cognizant of the potential statistical shortcomings given our admittedly small observed population.

**Figure 5:**
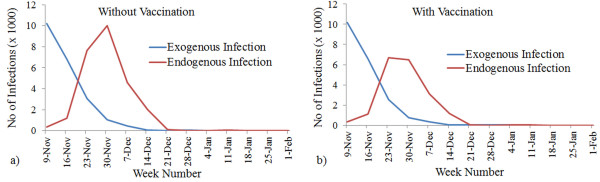
**Number of infections per week.** The number of exogenous and endogenous infections per week without vaccination (left), and with vaccination (right) over the course of 100,000 runs.

Figure 5 shows the number of exogenous and endogenous infections for two baseline scenarios varying the treatment of vaccination. The endogenous cases require exogenous infections to begin transmission through the network. The lag in response between the exogenous and endogenous curves is therefore expected. Because the exogenous pressure diminishes to nearly zero by January, the endogenous infections also disappear quickly. As there is no chance of reinfection in the model, and because recorded contacts virtually disappear over the Christmas break, the endogenous infections also diminish to nearly zero by January.

### Impact of overall network structure

Having established that the disease model and parameters are broadly consistent with the empirical observations regarding the H1N1 outbreak in the study population in Fall 2009, we used two scenarios with 100,000 realizations each (with parameters described in Methods Section and covering scenarios with and without vaccination) to evaluate the impact of network structure and dynamics on the spread of disease. Unlike most previous work in agent-based modeling, this study had recourse to detailed contact records containing not only high-fidelity temporal data, but also proximity estimates. By constraining our inquiry to a single contact criteria and ILI, we leveraged the strength of our dataset to investigate the impact of contact network structure and contact duration on the spread of a specific disease. Because of our large-scale Monte Carlo ensembles, we believe that the variations in the underlying H1N1 model data have been well explored; therefore, we expect that heterogeneity in the results of the simulations to be dominated by the impact of network structure and contact duration rather than simulation artifacts.

#### Depth of infection

**Figure 6:**
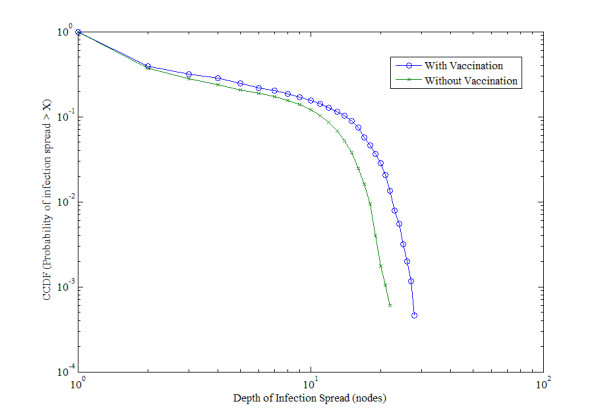
**Depth of infection spread.** Depth of infection spread for scenarios with consideration of vaccination (x’s) and without consideration (o’s).

Because the duration of contacts is characterized by an approximate power law relationship for much of its span (Figure 2b), it seems reasonable to hypothesize that the depth of infection spread will follow a similar trend. This presumes a highly connected network where duration of contact is the dominant parameter. Considering the final network connectivity plot shown in Figure 2c, this is a reasonable – but not entirely justifiable – assumption as subnets are likely to exist. The CCDF of the depth of infection spread is shown in Figure 6.

In both cases, the plot seems to generally follow a classic small-world network heavy-tailed power law distribution [21]. The depth of infection spread is slightly less in the vaccinated case, and seems to be asymptotic to a lower depth of infection. The overall shape is not surprising as it reflects the underlying structure of the network. However, it is worth noting that the figure shows the probability of an infection reaching at least a specific depth, normalized by the number of infections in that condition, so that the total number of infections represented by the vaccinated case is actually much lower than in the non-vaccinated case. This further implies that while vaccinating a fraction of the population can lower overall incidence rate, the pathogen can still penetrate the network to nearly the same degree over unvaccinated links.

#### Infection impacts

Before analysing impacts, it is necessary to establish appropriate metrics for measuring network connectivity. Table 1 shows correlation metrics (ρ) and *p* values for Pearson and Spearman correlations between likelihood of infection and the four measures of centrality introduced in Metric Section for contact networks structure, for baseline simulations with and without vaccination. Both tests were employed to highlight the differences in measures which are rank (Spearman) but not linearly (Pearson) correlated with infection probability, shedding light on the nature of the interaction.

**Table 1:** Correlations between centrality measures and probability of infection in baseline simulations

	*Ignoring H1N1 Vaccination*				*Considering H1N1 Vaccination*			
	**Pearson**		**Spearman**		**Pearson**		**Spearman**	
	**ρ**	**p**	**ρ**	**p**	**ρ**	**p**	**ρ**	**p**
Betweenness	0.172	0.316	0.245	0.149	0.110	0.522	0.239	0.160
Degree	0.415	0.012	0.292	0.084	0.296	0.080	0.258	0.128
TD	0.514	0.001	0.744	<0.001	0.344	0.040	0.519	0.001
LTD	0.740	<0.001	0.744	<0.001	0.503	0.002	0.519	0.001

Table 1 suggests that, in our experiments, betweenness centrality fails to capture the ∈s of network structure enhancing infection risk, as both experiments produced weak correlations with non-significant p-values for both Pearson and Spearman tests. Degree centrality has similar shortcomings, producing only one significant result with a moderate correlation and p = 0.012 using Pearson’s test, which is counterbalanced by the non-significant correlation using Spearman’s test. However, both TD and LTD centralities produce significant correlation results for the non-vaccinated case for both correlation coefficients, and moderately (TD) or very (LTD) significant correlations for the vaccinated case. We further infer that LTD is a more appropriate measure than TD because of the higher ρ in the Pearson case. While TD and LTD are both equivalently rank correlated under Spearman’s metric, LTD better satisfies the linearity assumptions in Pearson’s metric. We further explore LTD in Impact section of internal network structure using regression. Because the variation correlates with a network structure metric, we infer that the heterogeneities in overall infection rate of individuals across all simulations arise from the network structure or related parameters, consistent with the findings of [6].

While the correlations between time degree and probability of infection remain significant in the simulations that included vaccination information, the degree of correlation diminishes. This result is not surprising, as vaccination has a direct impact on the likelihood of infection, which goes to zero in the model regardless of the individual’s network connectivity. This observation is interesting for two reasons: first, it demonstrates that independent variation in infection likelihood diminishes the impact of network structure; and second, that even in the face of a highly non-linear, but not universal, disturbance (not all nodes are vaccinated), the underlying impact of network structure remains significant.

Student’s *t*-test was applied for each centrality measure to test the null hypothesis that those reporting and not reporting vaccination held identical mean centrality values. As it is shown in Table 2, p-values returned for the tests were all more than 0.7, indicating that the hypothesis of equal means could not be rejected. This further suggests that participants did not base vaccination decisions on their centrality and that differences between the vaccinated and non-vaccinated correlations in Table 1 are not due to a selection bias of high or low centrality participants opting for or against vaccination.

**Table 2:** **p-values resulting from applying Student’s** ***t*-test to test the hypothesis of equal means centralities for those reporting and not reporting vaccination**

	**Betweenness**	**Degree**	**TD**	**LTD**
p-Value	0.93	0.723	0.721	0.911

#### Transmission impacts

While network structure impacts the population spread of a pathogen due to its strong effect on infection risk, it also changes risk of transmission given infection. Table 3 shows the correlations between an agent’s centrality and the average number of secondary infections caused by that agent per episode of infection of that node. The results are generally similar to the infection risk correlations reported in Table 1, as expected because contact networks are generally undirected graphs. Traditional network measures (betweenness and degree centrality) still exhibit weak and non-significant correlations. By contrast, TD and LTD centralities exhibit stronger and consistently statistically significant correlations. As with risk of infection, when H1N1 vaccination is considered, the correlations are lowered. These results generally confirm those in [6] and extend them by adding the LTD metric.

**Table 3:** Correlations between network measures for a node and number of secondary infections caused by that node per each time it is infected

	*Ignoring H1N1 Vaccination*				*Considering H1N1 Vaccination*			
	**Pearson**		**Spearman**		**Pearson**		**Spearman**	
	**ρ**	**p**	**ρ**	**p**	**ρ**	**p**	**ρ**	**p**
Betweenness	0.184	0.283	0.244	0.151	0.139	0.420	0.253	0.137
Degree	0.399	0.016	0.300	0.076	0.302	0.073	0.296	0.080
Time Degree	0.665	<0.001	0.895	<0.001	0.472	0.004	0.615	<0.001
Log Time Degree	0.802	<0.001	0.895	<0.001	0.590	<0.001	0.615	<0.001

Finally, significant correlations between likelihood of infection and both TD and LTD centralities were maintained for those Monte Carlo ensembles in which behavior change was assumed to limit infection transmission to the asymptomatic period. The persistence of the correlations in this extreme case suggests that even in the presence of strong behavioral change on the part of symptomatic infectives themselves, duration-based measures are likely to remain important indicators of infection risk.

### Impact of internal network structure

**Figure 7:**
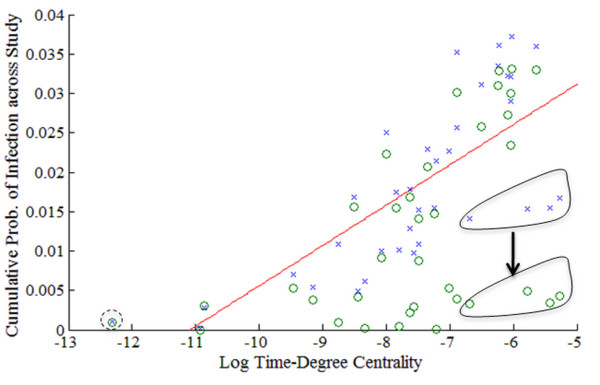
**Impact of LTD on endogenous infection probability.** Impact of a node’s log-transformed TD centrality (LTD) and immunization on endogenous infection probability. Results from two simulation scenarios are shown: One where the vaccination effect is considered (x’s), and another where this effect is ignored (o’s). The red line indicates the least squares fit for the case without vaccination. Dashed lines represent outliers; solid lines denote a single identified subnet.

While our number of participants is insufficient to make strong generalizations about the impact of internal network structure on the transmission of infection, the simulations based on the data can illustrate effects that may be observed in larger networks. The first and most apparent is the correlation between log time degree and the risk of both infection and transmission. The impact of network structure and vaccination on endogenous cumulative infection probability can be illustrated by plotting LTD against the cumulative likelihood of infection for both scenarios, as shown in Figure 7, with a least-squares regression line for the non-vaccinated, log time centrality data.

Although LTD centrality only offers an approximation of the likelihood of infection, the log-linear regression suggests a strong dependence on network structure in the absence of other effects. People with larger LTD centrality are linearly more likely to get infected, indicating a degree of predictive power. To fully verify this hypothesis requires a more rigorous statistical treatment and a larger participant population. This relation has limited predictive power because the probability of infection and therefore the slope of the line depend not only on the LTD centrality, but the parameters of the disease, and individual risk factors, which may be difficult to derive or collect in practice. The most we can conclude is that people with larger TD centrality will have a measurably increased risk of infection, with all other factors held equal.

The graph also indicates that there exists an LTD centrality below which it is impossible to become endogenously infected (near −11). Our own simulated data refutes this, as even the least connected individuals had non-zero endogenous infection counts. However, the *x* intercept could be usefully interpreted as the centrality below which infection probability is negligible. Although we have insufficient data to confirm this, a logical hypothesis to draw from Figure 7 is that the overall behavior of infection rate and LTD is sigmoidal with a linear central region and asymptotic saturation zones beyond which lower LTD is unlikely to confer protection, and higher LTD is unlikely to enhance risk. This would be an interesting avenue of future research given larger longitudinal datasets.

There are two primary sets of outliers in Figure 7: those who had very low centralities and did not often get infected in simulation (dashed line), and the main office staff who formed a semi-isolated subnet (solid lines). The office staff subnet has high mutual time degree but limited exposure to the rest of the network, generating relatively lower endogenous infection probabilities. This effect is multiplied by their high vaccination uptake, with 3 of the 4 members receiving H1N1 vaccination, ensuring herd immunity for the entire subnet.

**Figure 8:**
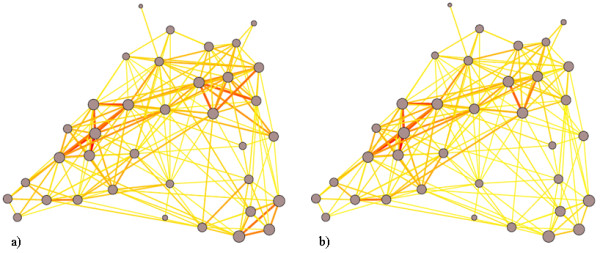
**Infection transmission across network.** Representations of infection rates across the network without **(a)** and with **(b)** vaccination. Node size represents participant LTD, node edge color and width represent infection events.

To better visualize the impact of network structure and vaccination, we created a network graph that shows the participants’ log degree centrality as the size of the node, and the number of infectious events between those nodes as the width and color of the line between them. Figure 8a represents infection events without vaccination and Figure 8b represents infection events with vaccination status taken into account.

Several qualitative observations can be made from Figure 8. The first is that while many participants experienced relatively high infection counts, the most concentrated infections occur between subnets of high LTD participants. There are three subnets in particular, in the middle-left, upper right and lower right hand portions of the graph. The middle and upper subnets (corresponding to different graduate labs) are bridged by a high centrality individual while the lower subnet (corresponding to the office staff) remains relatively isolated. In the vaccinated graph the middle subnet retains much of its topology while the upper subnet shows a lower infection count as one of the high LTD nodes received vaccination. The lower subnet has its infection rate fall dramatically, as noted above.

The impact of local effects on the office staff subnet is particularly pronounced, likely due to their strong intra-subnet and weak inter-subnet connections. If this was the case, then endogenous infections should be preferentially transmitted between members of the main office subnet, and not with members outside their subnets. Table 4 shows the relative infection rates of the main office subnet.

**Table 4:** Fraction of transmission which happened between the main office subnet and any other member of the network

	Member 1	Member 2	Member 3	Member 4
Fraction of transmission from/to outside members	6.88%	6.54%	17.89%	0.56%

It is clear from the table that the vast majority of the infectious events occur by passing infection inside the office subnet. When one subnet member is infected exogenously, other members stand a much higher chance of infection, given their very large subnet LTD. The main office has a relatively small chance of contracting an infection from the rest of the network, and a much higher chance of contracting an infection within their own subnet. This forces the infection rate for LTD down as shown in Figure 7 because – although their LTD is relatively high – their isolation offers them some protection. While the phenomenon itself is fascinating, it is unclear given our small sample size to what degree isolated networks exist in the population, and to what degree they drive or inhibit the spread of infection through a population.

## Discussion

The work in this paper builds on approaches explored in important past contributions. These include individual [4,16] and aggregate [22] transmission models using manually self-reported contact data that distinguish multiple contact intensities; and recent contributions also include simulations on sensor-based contact micro-data but using highly stylized model parameter values not tied to any specific pathogen [6,7,23]. As the first contribution to combine epidemiologically grounded transmission modeling of a specific infection with electronically collected contact micro-data during an outbreak, the work described here offers some important methodological lessons. Study findings demonstrate the benefits of leveraging multiple data sources during an epidemic – including electronically collected data – into simulation models. This methodology highlighted the importance of contact duration variability in shaping the spread of an airborne pathogen, beyond [6] by introducing the concept of log time degree motivated by previous observations of intercontact dynamics. While the small sample sizes involved greatly limit the strength of the conclusion, the study is also notable in supporting the consistency of published parameter estimates for H1N1 models [11] with observed transmission patterns in Saskatoon.

### Limitations

While the results presented here constitute a clear methodological contribution to study of outbreaks, our techniques and data have implicit limitations. First and foremost, our findings are only demonstrated for the sub-population under observation, and the particular disease dynamics of H1N1. Other researchers [24,25] have found differing impacts for other simulated populations and different disease dynamics.

· **Individual Behavior:** We assumed that the contact dynamics of an individual within the dataset was independent of their own and their contacts’ infection status. While we examined an alternative scenario that examined the impact of assuming extreme behavior change following the appearance of symptoms, this scenario does not capture important direct and indirect effects, including the spread of risk perception, social distancing, and proactive protective measures among the study population.

· **Data Set Size:** Consistent with other electronic contact monitoring studies [12,13,15], the contact network data used in this study is drawn from a small and specialized study population, imposing a strong selection bias. While the data set here had a modest number of participants when compared to [6] we collected data over substantially longer study duration.

· **Exogenous Contacts:** Lacking significant data on external contacts, we were forced to assume a uniform exogenous infection pressure for all individuals based on population-level statistics, which may mask potentially important diversity in vulnerability to external infection.

· **Equivalence of Place:** In adapting model parameter estimates from the published H1N1 model, we made the assumption that the populations experienced similar basic reproductive numbers – despite differences in population density and demographics.

Despite such limitations, we believe that the findings presented here emphasize the utility of combining simulation modeling and ambulatory data collection, and highlight the considerable value to be gained in model building, decision making and operational prioritization by adding duration-based measures into surveillance. The findings also underscore the importance of future studies using broader and more diverse study populations, improved understanding of exogenous contact patterns and behavior change, and refined simulation models.

### Contributions

Many previous contributions have highlighted heterogeneity in population contact rates. Particularly pronounced heterogeneity has been observed in sexual contacts, for which contact rates appear to obey power law distributions [25]. Past modeling efforts have emphasized the importance of such heterogeneity – particularly contact diversity [26] and concurrency [27] – in the population spread and endemic persistence of infection. By contrast, prominent past studies using self-reported casual contact patterns [4] found little evidence of power-law diversity in contact frequency, but study design prevented examinations of heterogeneity in contact durations. While our results support previous long-term observations using automated data collection [15] of power-law heterogeneity in contact duration, and its impact on disease spread [6,7], our modeling results appear to be the first to use a model grounded in outbreak data to suggest that such heterogeneity in contact duration could be of equal or greater importance to infection spread as heterogeneity in contact frequency. In light of the apparent significance of the empirical contact durations for infection spread, we recommend that other simulation models carefully consider and test assumptions made regarding contact duration and node degree.

The importance of contact duration in modeling infection spread reflects two key roles that it plays in infection spread. First, it is a predictor for infection risk. Our results suggest significantly stronger associations between infection risk and duration-related network measures than are found using traditional centrality measures, confirming the results presented by [6]. We have extended these results to provide a measure of predictive power by noting that social networks tend to scale as truncated power law or exponential distributions [21], and proposing that the logarithm of total contact duration better represents the risk than does duration itself. The advantage offered by duration-related measures is particularly significant in light of recent contributions highlighting strong associations between degree centrality and risk of infection in contact traced individuals [28], and the importance of degree centrality for timing of illness [29]. The correlations in Table 3 and diagrams in Figure 8 further suggest that, when infected, an individual with higher contact duration is likely to infect more individuals. This relationship exhibits a higher degree of correlation than is seen between secondary infections and traditional centrality measures. The stronger correlations involving contact duration are of particular interest given degree centrality’s proposed role as a risk marker for prioritizing prophylaxis [30]. Both findings suggest that use of contact-duration-based measures may provide additional benefit when prioritizing vaccination, contact tracing or prophylactic treatment.

The desirability of contact-duration measures for modeling, decision-making, and operational prioritization has broader health surveillance implications. Although it is possible to collect time-weighted duration information using traditional self-reporting, results of prominent studies employing self-report have questioned the feasibility of imposing the additional requisite bookkeeping burden, further suggesting that automated mechanisms may be required [4]. Our experience with Flunet suggests that many people have limited tolerance for the tedium of recollecting contacts and recording even a rank ordering of those contacts based on contact duration. In Flunet, participant compliance with requested self-reporting of their 5 most common weekly contact durations was just above 25%. Given the apparent value that time-duration information offers for simulation modeling and decision-making, these study results suggest that electronically collected contact information can offer a particularly valuable role in complementing existing data sources in epidemiological surveillance and investigation.

While other contributions [6,7] have demonstrated the power of combining micro-contact data with simulation models, to the best of our knowledge, we are the first to propose our novel “Groundhog Day” methodology, which leverages not only the micro-contact data, but empirically grounded time-varying models of endogenous pressure and Monte Carlo agent-based simulations. While this technique could encounter significant computational challenges when scaling to population-level modeling – even if the contact data were available – it provides an excellent methodology for probing and analysing risks of at-risk target populations such as those in care homes or in college dormitories.

### Future research directions

While the contributions of this paper are largely methodological – pertaining to the use and utility of micro-contact data in monitoring and modeling outbreaks – we have made several observations based on our data that merit further examination in the future using larger datasets. First, we noted a regressive fit between LTD and infection risk. With a larger study population, one could utilize standard epidemiological statistical techniques such as logistic regression to disambiguate the relative risk of LTD when compared to other factors such as age, gender, occupation, or socio-economic status. We also hypothesized that the actual relationship between LTD and infection risk would be characterized by a sigmoidal or similar function, with asymptotic minima, corresponding to a baseline chance of contracting a pathogen from the environment and maxima, corresponding to the point at which additional links confer little additional risk, as infectious exposure is almost guaranteed. Substantially larger datasets would be required to probe the extremes of the distribution. Finally, we posited that the internal network structure can impact the probability of infection, by comparing risk between two connected and one isolated subnet of high LTD participants. The isolated subnet received relatively few endogenous infections from the rest of the network, but suffered high mutual infection rates. As the workplace conditions of the main office staff is more in keeping with traditional Western work habits (predominantly defined schedules, and location) than graduate students (predominantly undefined schedules, roving locations), it may be that these isolated subnets could drive pathogen transmission elsewhere in society more than currently appreciated. Larger, and ethnographically broader, datasets will be required to properly investigate this hypothesis. While we have made strong methodological contributions to the study of pathogen spread, the detailed questions and hypotheses generated during our analysis may have a more significant long-term impact.

## Conclusions

In this work we have presented the results of combining a micro-contact dataset and a population health data and simulation modeling methodology – termed a Groundhog Day system – for the study of the impact of contact dynamics on the spread of H1N1 influenza through a small study population during the 2009 flu season. Our results validated the transmission model, in providing close agreement with observed infection rates within the study population – as gathered with surveys. We leveraged the temporal span of our data to derive a risk metric – log time degree – which appears to correlate with both risk of being infected and risk of infecting given infection, all other factors held equal. The methodology described here is an important step in leveraging both personal and scientific computing for the study of infectious disease.

## Competing interests

The authors declare that they have no competing interests.

## Authors’ contributions

MH collected the contact data, implemented and ran the simulations, performed analysis and contributed text to the paper, NO provided the initial experimental and simulation design and contributed to the paper, KS provided the initial data collection design, performed analysis and contributed to writing of the paper. All authors read and approved the final manuscript.

## Pre-publication history

The pre-publication history for this paper can be accessed here:

http://www.biomedcentral.com/1472-6947/12/35/prepub
